# HIV and Early Treatment Outcomes Among Women With Cervical Cancer Treated With Concurrent Chemoradiation in Tanzania

**DOI:** 10.1200/GO.22.00441

**Published:** 2023-09-22

**Authors:** Alita Steven Mrema, Mamsau Ngoma, Chacha Josiah, Emmanuel Lugina, Nanzoke Mvungi, Magreth Paul, Evelyne Mkuchika, Emmanuel Nundu, Salama Khamisi Iddy, Eulade Rugengamanzi, Yokobeth Mtambo Vuhahula, Faraja Christopher Kiwanga, Charles Wood, Julius Mwaiselage

**Affiliations:** ^1^ Clinical Oncology Department, Ocean Road Cancer Institute, Dar es Salaam, Tanzania; ^2^ Muhimbili University of Health and Allied Sciences, Dar es Salaam, Tanzania; ^3^ Louisiana State University Health Sciences Center, New Orleans, LA

## Abstract

**PURPOSE:**

Cervical cancer (CC) is the leading malignancy in Tanzania. Low-income countries are faced with double epidemics of HIV and CC. This study aimed to investigate how HIV and cancer stage at diagnosis affect early treatment outcomes among women with CC treated with concurrent chemoradiation in Tanzania in the highly active antiretroviral therapy era.

**MATERIALS AND METHODS:**

This was a prospective cohort study of patients newly diagnosed with CC at the Ocean Road Cancer Institute from November 2019 to January 2020. The tumor response was assessed using RECIST 3 months post-treatment. The tumor response was categorized as a complete or partial response according to the ultrasound and pelvic examination findings. The univariate and multivariate logistic regression explained the relationship between several covariates (age, stage, HIV status, equivalent dose in 2 Gy fractions, chemotherapy cycles, and treatment time) and treatment response.

**RESULTS:**

A total of 102 patients with CC were included in this study at baseline. After adjusting for other covariates, only completion of treatment within 56 days (odds ratio [OR], 9.23; 95% CI, 1.53 to 55.85; *P* = .016) and receiving at least three cycles of cisplatin (OR, 5.6; 95% CI, 1.47 to 21.34; *P* = .012) were significantly associated with complete tumor response. HIV status was not significantly associated with complete tumor response (OR, 1.534; 95% CI, 0.424 to 5.545; *P* = .5144).

**CONCLUSION:**

Early treatment response was independent of HIV status. With wide coverage of anitretroviral therapy, patients with HIV can receive radical treatment and have the same early outcomes as their HIV-negative counterparts.

## INTRODUCTION

Cervical cancer (CC) disproportionately affects low-income countries where approximately 84% of all cases reside.^1^ Sub-Saharan Africa (SSA) has the highest CC burden compared with the rest of the world.^2^ The highest incidence and mortality of CC in SSA are attributed to barriers to accessing cancer services such as screening and treatment.^3^ The majority of patients present to the hospital with late stages of the disease.^3^

**CONTEXT**

**Key Objective**
This study aimed to investigate how HIV status and cancer stage at diagnosis affect early treatment outcomes among women with cervical cancer (CC) treated with concurrent chemoradiation in Tanzania in the highly active antiretroviral therapy (HAART) era.
**Knowledge Generated**
Findings showed that women living with HIV with CC have a comparable early treatment response with HIV-negative women with CC in the HAART era probably because of immune restoration.
**Relevance**
Treatment response may be used as a proxy indicator for survival in the HAART era.

Human papillomaviruses (HPVs) are associated with almost all CCs.^4^ HIV infection is associated with persistent HPV infection and an increased risk of precancerous lesions.^5^ The prevalence of genital high-risk HPV among adult women in Tanzania is estimated to be 20%.^6^ The adult HIV prevalence in Tanzania is estimated to be 4.9%.

Concurrent chemoradiation (CCRT) is the mainstay of treatment for locally advanced CC. This treatment modality improves local tumor control and survival. The role of platinum-based chemotherapy is to increase tumor radiosensitivity and eliminate micrometastasis.^7^ However, the treatment of HIV-positive patients with CC is challenging because of the interplay of several factors such as immunocompromised status, drug interaction, the risk of opportunistic infections, and other psychosocial factors.^8^

The overall mortality rate for HIV-infected patients with cancer is higher than that for noninfected patients.^9^ The excess mortality is partly due to AIDS, but an adverse effect specifically on cancer outcomes is biologically plausible if an intact immune system helps control cancer after treatment.^10^ In a study of patients with CC in Botswana, most patients presented with advanced-stage tumors. Although HIV-infected and HIV-uninfected women were equally likely to have a complete tumor response to initial treatment, HIV infection was associated with a doubling in overall mortality and more than 97% of deaths were attributed to cancer.^11^

There is a paucity of data in Tanzania concerning the effect of HIV on early CC treatment outcomes in the highly active antiretroviral therapy (HAART) era. This study seeks to explore the effect of HIV infection on tumor response among patients with CC receiving radical treatment at the Ocean Road Cancer Institute (ORCI).

## MATERIALS AND METHODS

ORCI is the national referral hospital located in Tanzania. It receives approximately 5,000 cancer cases per year from all over the country. CC accounts for approximately 60% of all cases. The hospital is equipped with four radiotherapy machines (two linear accelerators [LINACs] and two cobalt machines) plus two high-dose rate two-dimensional (2D) intracavitary brachytherapy (ICBT) units.

### Study Participants

This was a prospective cohort study that enrolled participants who were histologically diagnosed with CC from November 2019 to January 2020. All participants were treated at ORCI with CCRT. Patients with unknown HIV status were counseled to do HIV tests, and those who declined to test were excluded from the study. Participants with HIV had their baseline CD4 taken before treatment and were assessed by a physician if they were on antiretroviral therapy (ART) or not. If participants were not on ART, they were counseled to start treatment. The staging was performed through a clinical examination, chest x-ray, and abdominal pelvic ultrasound (USS).

Cross-sectional imaging was not used because of affordability and access issues. The 2009 International Federation of Gynecology and Obstetrics staging system criteria were used to stage the tumors.

Participants provided written informed consent. The study was approved by the ethics committee of ORCI. Treatment consisted of CCRT followed by ICBT. Total radiotherapy doses were calculated by using the equivalent dose in 2 Gy fractions (EQD2) formula. Tumor size was assessed before treatment and 3 months post-treatment through a gynecologic examination and an abdominal pelvic USS. Participants were categorized into complete response (CR) and partial response (PR) according to the RECIST. The CR was defined as the disappearance of all target lesions, whereas PR was defined as at least a 30% decrease in the sum of diameters of target lesions, taking as reference the baseline sum diameters as per abdominal pelvic USS findings. Only participants treated with curative intent were included in the final analysis.

### Treatment Protocol

The standard treatment protocol included external beam radiotherapy (EBRT) dose ranging from 45 to 50 Gy using a LINAC machine with conformal three-dimensional (3D) planning and a 15-MV energy and four-field (Box) technique for those who were treated with curative intent. Patients with a history of hysterectomy before radiotherapy were prescribed 45 Gy to minimize the dose to the bowels, whereas those without hysterectomy were prescribed 50 Gy. A hypofractionation schedule of 40 Gy in 16 fractions was used for participants who had poor performance status, hydronephrosis, and a deranged renal function test. EBRT was followed by a 20-25 Gy high dose rate after loader 2D ICBT with Cobalt 60 (Co-60) source to point A. ICBT was delivered in three fractions, each of 8 Gy using tandem, and ring applicator was given. Participants who had a tumor larger than 4 cm after EBRT were not given brachytherapy after completion of EBRT; instead, they received a boost EBRT dose of 14 Gy in seven fractions to a total of 64 Gy to the central tumor. Participants who were treated with palliative intention received radiotherapy doses ranging from 10 to 30 Gy by using Co-60 teletherapy machine.

Radiotherapy doses with an EQD2 of more than 79 Gy to point A were considered curative. This was a sum of the EBRT and ICBT doses.

For analysis purposes, guideline-concordant treatment was defined as radiotherapy treatment with an EQD2 of more than 79 Gy together with at least three cycles of concurrent chemotherapy. EQD2 doses ranging from 71 to 79 Gy were considered minimally adequate, whereas those below 71 Gy were considered inadequate. The early disease was defined as stage IB-IIA, and the advanced disease was defined as stage IIB-IVA. Para-aortic lymph node fields were not used since there were no participants with radiologic confirmation of para-aortic nodes.

Concurrent cisplatin (40 mg/m^2^) was given once a week for up to five cycles unless there was impaired renal function (creatinine clearance ≤50 mL/min), hematologic toxicity, poor performance status, and financial constraints. In the case of renal impairment, carboplatin was given once a week during EBRT with dose calculation of the area under curve 2.

### Statistical Analysis

The continuous variables were described using the mean and standard deviation for normally distributed variables or the median and range for non-normally distributed variables. Categorical variables were described using frequency and percentages. The chi-square tests were used to test the association between two categorical variables. Fisher's exact test was used if there was a violation of chi-square assumptions. The nonparametric Kruskal-Wallis test was used to compare the non-normally distributed variables (age of the patient, age at menarche, and age at first sexual intercourse) across HIV status. The univariate and multivariate logistic regression explained the relationship between several covariates and treatment outcomes (CR *v* PR). The following covariates were included in the logistic regression model: the age of the patient, stage of the disease, HIV status, EQD2, number of chemotherapy cycles, and treatment duration. Data were analyzed using SAS version 9.4 software (SAS Institute, Cary, NC).

## RESULTS

### Sociodemographic Characteristics of the Study Participants

A total of 101 participants were included in this study at baseline. However, 10 patients absconded treatment, and two died before treatment initiation. Of the remaining 89 patients, only 87 patients received curative treatment. Among 101 patients recruited in this study, 42 (41.6%) were HIV-positive. The patients with HIV were younger than the patients without HIV (median age of 45 years *v* 57 years; *P* < .0001). The distribution of alcohol use, cigarette smoking, and education level did not differ by HIV status (*P* > .05; Table 1).

**TABLE 1:**
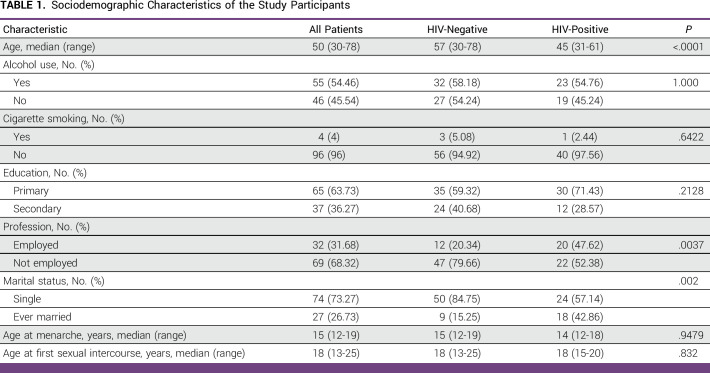
Sociodemographic Characteristics of the Study Participants

### Clinical-Pathologic Characteristics of the Study Participants at Baseline

There was no difference in the stage at presentation according to HIV status (*P* = .8339).

Eighty-nine patients received radiotherapy treatment. Of these 89 patients, 87 patients were treated with a curative intention, whereas two patients were treated with palliative radiotherapy. The median EBRT dose was 50 Gy (range, 10-64). Of 89 patients, most patients (84, 93.33%) received more than 45 Gy of EBRT. Of 74 patients who received ICBT, 73 (98.65%) received at least ≥18 Gy of ICBT. Only one patient who was HIV-negative did not receive the ICBT of brachytherapy because of COVID-19 pandemic interference. On the calculation of EQD2, 73 (82.02%) patients received the recommended dose of radiation therapy (EQD2 >79 Gy), whereas 16 (17.98%) received <79 Gy of radiation therapy. The received radiotherapy dose did not differ by HIV status (*P* = .1629).

The chemotherapy cycles received did not differ according to the HIV status (*P* = .2949).

Overall, most patients (80, 91.95%) received treatment within the recommended treatment duration (<56 days), whereas the remaining few (7, 8.05%) received a longer treatment (more than 56 days). However, treatment duration did not differ across HIV status (*P* = .4538). Of 87 patients who were planned for curative treatment, more than half (51, 58.62%) did not receive guideline-concordant treatment. Indexed by HIV status, still, most of the patients with HIV (27, 71.05%) did not receive guideline-concordant treatment, whereas in the HIV-negative group, the ratio of those who received versus those who did not receive guideline-concordant treatment was approximately equal.

The HIV subcohort had a median CD4 count (507 cells/μL; range, 17-1,054). Nearly all 41 (97.62%) patients were on ART at baseline except one patient who refused to take ART (Table 2).

**TABLE 2:**
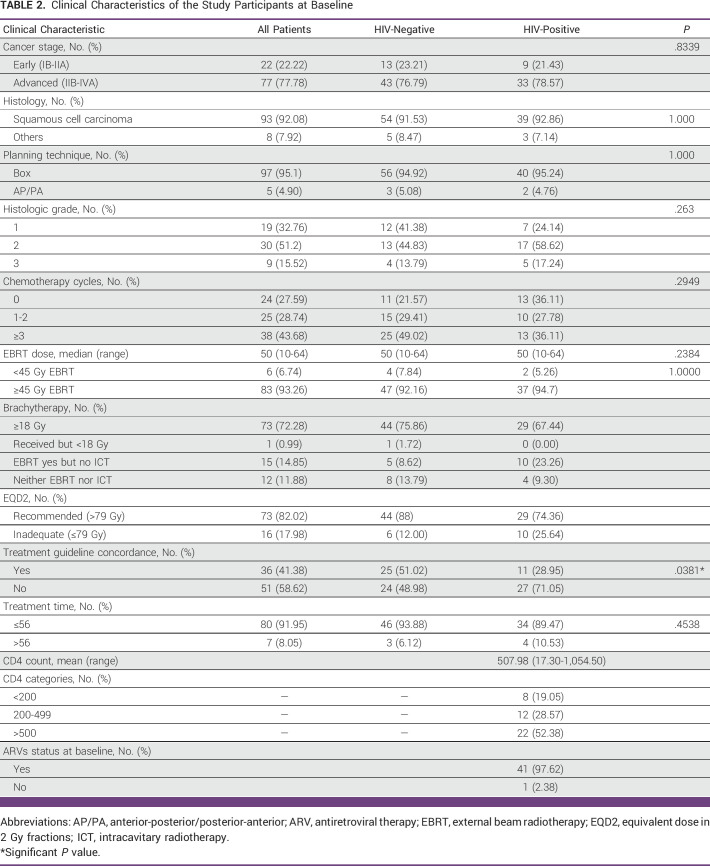
Clinical Characteristics of the Study Participants at Baseline

### Factors Associated With Treatment Response Among Patients With Cervical Cancer

The univariate and multivariate analyses included only participants who were treated with curative intent. In the univariate analysis, the early stage of the disease, EQD2 >71.2 Gy, receiving at least three cycles of cisplatin, and completion of treatment within 56 days were significantly associated with CR. HIV status was not significantly associated with the CR (Table 3).

**TABLE 3:**
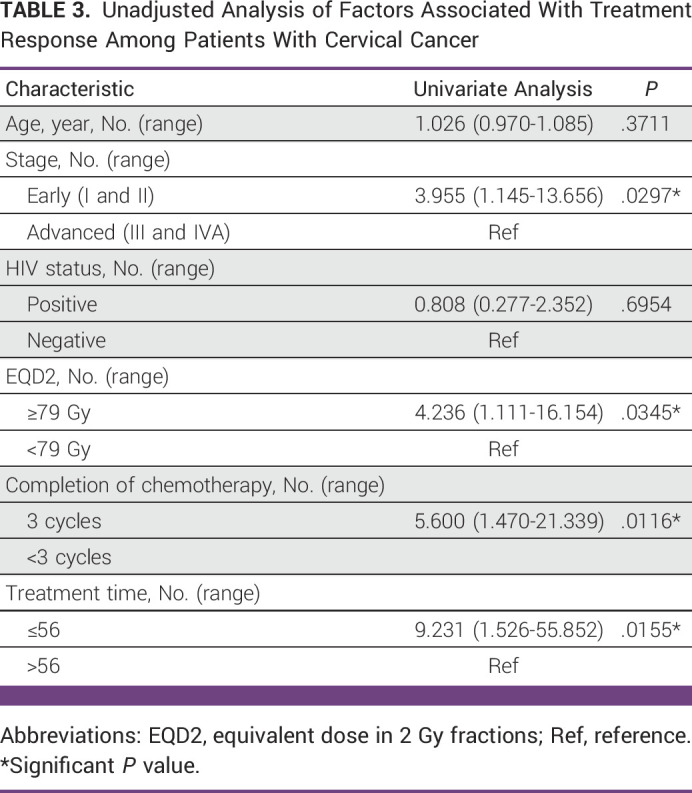
Unadjusted Analysis of Factors Associated With Treatment Response Among Patients With Cervical Cancer

In the adjusted analysis, patients who completed treatment within 56 days were 10.24 times more likely to achieve a complete tumor response compared with those who completed their treatment in more than 56 days after adjusting for other covariates (*P* = .022). Similarly, patients who received at least three cycles of cisplatin were 4.48 times more likely to achieve complete tumor response compared with those who received less than three cisplatin cycles (*P* = .047; Table 4).

**TABLE 4:**
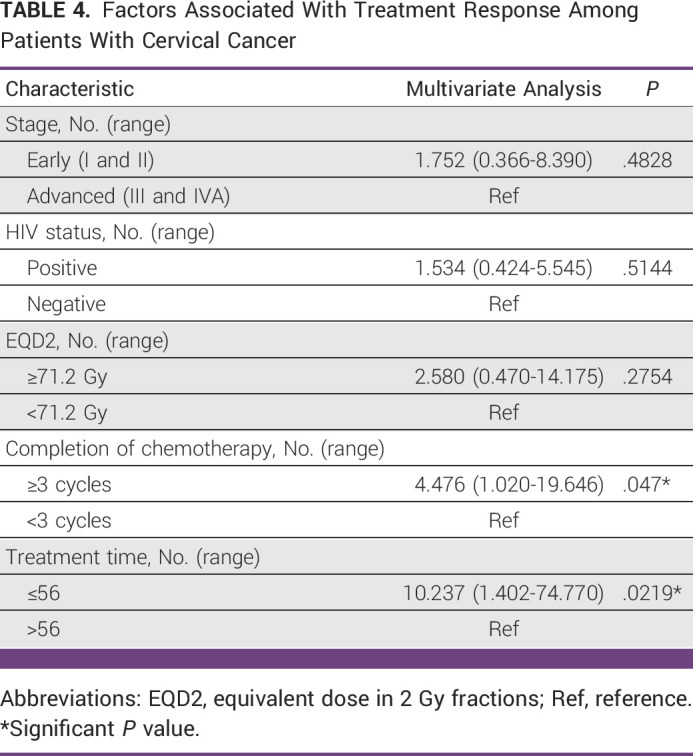
Factors Associated With Treatment Response Among Patients With Cervical Cancer

## DISCUSSION

In this study, participants without HIV and with HIV with CC were comparable in many sociodemographic and behavioral characteristics except for age, profession, and marital status. Specifically, patients with HIV were younger, with a high proportion being married. The evidence that HIV infection accelerates the progression to invasive CC might explain the young age at the onset of CC in people living with HIV.^12^

Many of the clinical characteristics were comparable between patients with HIV and without HIV with cancer. Approximately 75% of the participants presented with advanced stages of the disease (stages III and IV), highlighting the challenges in access to cancer care in SSA. About 10% of patients absconded from treatment because of myths against radiotherapy treatment and a preference for traditional medicine. A study in Northern Tanzania found that a majority of participants used traditional medicines because of lower cost, fewer side effects, ease of access, and perceived safety and efficacy.^2^

In the current study, the imaging modality used to assess the tumor response was USS. According to the revised RECIST guideline (version 1.1), USS is not useful in the assessment of lesion size. USS cannot be reproduced in their entirety for an independent review at a later date, and, because they are operator-dependent, it cannot be guaranteed that the same technique and measurements will be taken from one assessment to the next. The recommended imaging modality is either MRI or computed tomography scan.^13^ The high cost restricts the use of these cross-sectional imaging modalities for routine surveillance, in low- and middle-income countries.^14^ Moreover, there are very few studies that have investigated the role of USS in monitoring CC response to CCRT.^14^ Some studies have shown that USS can detect changes in the echogenicity and heterogeneity in the cervical tumor after CCRT and consequently showed its potential in characterizing early tumor responses, as early as 1 or 2 weeks^14^

The majority of participants received the intended curative treatment. However, more than half of the patients did not receive guideline-concordant treatment, and the proportion was much higher (>70%) in the participants with HIV. Reasons for treatment guideline discordance were inadequate radiotherapy doses as a result of brachytherapy incompletion. Disease progression in between treatments (development of a vesicle vaginal fistula), poor performance status, and big tumors after completion of EBRT led to ICBT omission. This finding is supported by the study from South Africa, whereby participants with HIV had higher rates of incomplete treatment because of inadequate radiation deficit caused by failure to receive brachytherapy compared with their counterparts without HIV.^15^

The index study has shown the use of at least three cycles of cisplatin, and completion of radiotherapy treatment was associated with more likelihood of attaining a complete tumor response. Although cisplatin has shown to have benefits when used together with radiotherapy, its optimum schedule and dosing require further review. In congruence with this study, three randomized trials reported that the use of CCRT was associated with superior survival and better treatment outcomes compared with the use of radiotherapy alone. Specifically, there was a 30%-50% decrease in the risk of death for patients who received CCRT.^16-18^

In this study, participants who were able to complete curative treatment within 8 weeks were more likely to achieve complete tumor remission. Similar findings from other studies showed better local tumor control and overall survival (OS) among patients who received definitive chemoradiation and completed it within 8 weeks.^19-21^ Approximately 90% of patients received radiotherapy treatment within the recommended time. There is geographical variation in average CC treatment time worldwide. However, a systematic review of CC management in SSA showed that the average treatment time was <8 weeks^22^ The upgrade of low-dose-rate brachytherapy to high-dose-rate brachytherapy machines in 2012 might explain the shortening of overall treatment time in the index study.

In the current study, there was no difference in early tumor response by HIV status among women with CC treated with CCRT. A study performed in Brazil by Ferreira et al^10^ also concluded that HIV was not associated with early treatment response, but instead, participants with HIV had a higher risk of relapse after achieving a CR and elevated mortality risk when the follow-up was more than 2 years from initial diagnosis. A systematic review reported that early treatment response is comparable between patients without HIV and with HIV with CC after treatment with CCRT; however, factors affecting treatment outcomes, such as treatment selection bias, pretreatment hemoglobin levels, and antiretroviral therapy administration, were not systematically accounted for.^23^ The comparable treatment outcomes in the index study could be attributed to very wide ART coverage (97.6%) and a high median CD4 count (507 cells/μL), indicating a better immune recovery among patients with HIV. This finding was supported by the prospective study performed in Botswana, which showed that differences in treatment outcomes of women with CC after CCRT were no longer significant when the HIV+ cohort was restricted to only those on ART.^24^

Cross-sectional imaging modalities could not be used because of affordability issues. The use of USS for staging might have precluded accurate determination of para-aortic and pelvic lymphadenopathy. In addition, toxicity data during treatment could not be captured.

In conclusion, to our knowledge, this is the first prospective study comparing early treatment outcomes between patients with HIV and without HIV with CC in Tanzania and individuals with HIV have the same early tumor response as their counterparts without HIV in the era of ART use. Although the sample size was small, it has provided highlights on early disease outcomes. Future studies will look into long-term treatment outcomes including relapse patterns, progression-free survival, and OS in this population.
